# Clinical and Histopathological Determinants for Kidney Allograft Survival in the Eurotransplant Senior Program (ESP) at the Time of Allocation

**DOI:** 10.3389/ti.2025.14153

**Published:** 2025-06-02

**Authors:** Tom N. Langer, Thorsten Wiech, Mercedes Noriega, Sergey Biniaminov, Tobias B. Huber, Lutz Fischer, Florian Grahammer, Malte A. Kluger

**Affiliations:** ^1^ III. Department of Medicine, University Medical Center Hamburg-Eppendorf, Hamburg, Germany; ^2^ University Transplant Center (UTC), University Medical Center Hamburg-Eppendorf, Hamburg, Germany; ^3^ Institute of Pathology, Nephropathology Section, University Medical Center Hamburg-Eppendorf, Hamburg, Germany; ^4^ Hamburg Center for Kidney Health (HCKH), University Medical Center Hamburg-Eppendorf, Hamburg, Germany; ^5^ HS Analysis GmbH, Karlsruhe, Germany; ^6^ Department of Visceral Transplantation, University Medical Center Hamburg-Eppendorf, Hamburg, Germany

**Keywords:** kidney transplantation, elderly, ESP European Senior Program, AI histopathology, machine learning

## Abstract

To address the shortage of organs for kidney transplantation, the Eurotransplant Senior Program (ESP) was established to enhance kidney allocation from elderly donors. This study aimed to evaluate post-transplant outcomes of deceased donor grafts and identify prognostic factors within the ESP population. We therefore analyzed patient data from 64 ESP recipients and their donors transplanted at our center between 2017 and 2022. Time-zero biopsies were analyzed using AI image analysis software for glomerular density and glomerulosclerosis. One-year patient and allograft survival rates were 96.9% and 85.9%. 5-year survival rate was 74.6%, as opposed to about 41.0% historically reported for patients on dialysis. Delayed Graft Function occurred in 29.7% of cases, with recipient coronary heart disease, BMI-disparities, and prolonged cold ischemia time as major predictors (*P* < 0.05). Histopathological analysis revealed that the degree of glomerulosclerosis and interstitial fibrosis and tubular atrophy (IFTA) were associated with graft failure in multivariable analyses (*P* < 0.05). Arteriolosclerosis (arteriolar hyalinosis) correlated with a higher risk for primary non-function (*P* < 0.05). The number of HLA mismatches was not significantly associated with graft outcome. Including prognostic baseline characteristics as well as histopathological AI analysis into individual allocation decisions during organ-acceptance process might improve allograft survival within the ESP and should prospectively be studied.

## Graphical Abstract

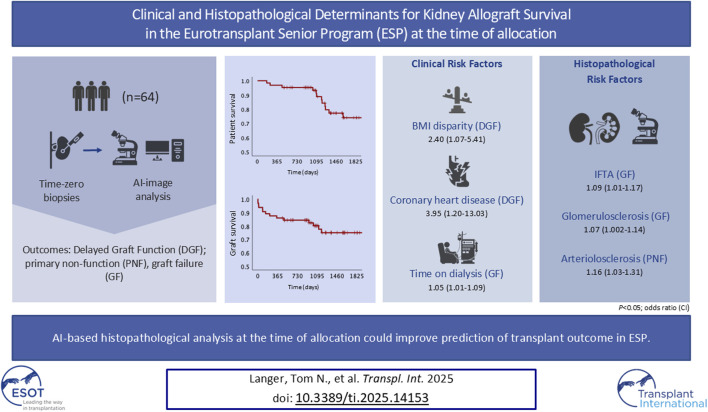

## Introduction

At present, kidney transplantation represents the only treatment option for patients suffering from terminal kidney failure that offers perspectives for prolonged survival and benefits for the quality of life. In response to the demographic changes, including the rising numbers of elderly patients with end-stage kidney diseases on the waiting list but persisting shortage of donated organs, Eurotransplant established the European Senior Program (ESP) for this group in 1999. The ESP allocates kidneys from deceased donors aged ≥65 years to elderly recipients ≥65 years of age who left the general kidney waiting list (ETKAS) for the benefit of significantly shorter waiting times. Its medical outcome is mainly based on minimizing cold ischemia time (CIT) by allocating organs locally, still based on blood group compatibility and waiting time. In contrast to the Eurotransplant Kidney Allocation System (ETKAS), the ESP does not include human leukocyte antigen (HLA) A-B-DR matching or specific immunological criteria. The latter have to be evaluated by the accepting centers, although inclusion of HLA-DR matching has recently been discussed [1]. Taken together, relevant reductions in waiting times for patients that otherwise might not even live up to their ETKAS-transplantation, as well as improved mortality rates among these elderly patients when compared to those continuing on dialysis, seem to be the major significant advantages of this program [2, 3].

Despite 25 years of experience with the ESP, selecting suitable organs from elderly donors remains a complex challenge due to the lack of extensive scientific studies identifying robust prognostic factors for satisfactory transplant outcomes. Frequently debated factors contain donor and recipient age, number of HLA mismatches, kidney re-transplantation, and body mass index (BMI) [1, 4–6]. Delayed graft function (DGF) is a significant prognostic indicator for graft survival and immunological response in ESP patients [4, 7–9]. Identifying modifiable risk factors for DGF could therefore contribute to improved outcomes in the future.

In this retrospective single-center study, we analyzed patient and graft survival in recipients of kidneys allocated via the ESP. Donor and recipient data were utilized to identify prognostic factors associated with kidney allograft survival and DGF. Furthermore, we evaluated whether the results of in-advance biopsies, that in our center are currently performed as time-zero analysis during transplantation, could potentially even further improve the prediction of the graft outcome when added to the aforementioned criteria, especially when their personnel- and time-sensitive processing could at least partially be automated. In addition, we aimed to review whether the ESP-recipients at our center in general still benefit from their transplantation.

## Material and Methods

### Study Design

From 1 September 2017 to 1 September 2022, 64 waitlisted recipients aged ≥65 years at the Hamburg University Transplant Center (UKE) received deceased donor kidneys via the ESP allocation algorithm. All renal allografts were obtained from donors after brain death, aged ≥65 years. Following the standard ESP criteria, HLA matching was not utilized during allocation. Induction immunosuppressive treatment consisted of basiliximab and steroids. Highly immunologically sensitized patients or patients with a high risk for DGF (e.g., longer CIT received thymoglobulin instead together with steroids. Maintenance immunosuppression included calcineurin inhibitors (mostly tacrolimus) and antimetabolites (mycophenolate mofetil or mTOR inhibitor) with or without steroids. From 2021 on, patients with low immunological risk were routinely placed on a steroid-free maintenance therapy from day eight after transplantation, following the HARMONY-study protocol [7].

### Data Collection

**FIGURE 1:**
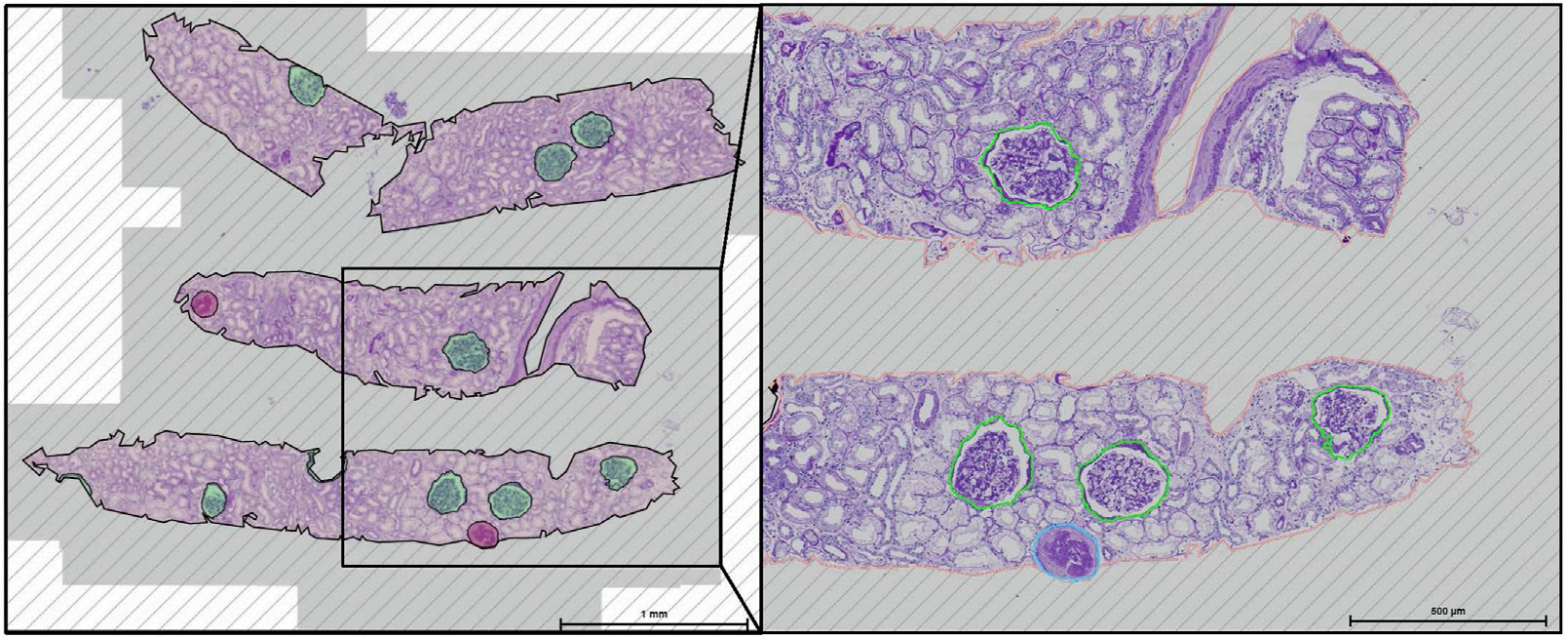
Deep learning-based glomeruli detection in HAS KIT from periodic acid Schiff stained kidney.

Donor data was extracted from Eurotransplant’s donor kidney reports. Recipient data was collected in a retrospective manner, utilizing the patient files and hospital discharge reports, with a minimum follow-up of 16 months. 18G-time-zero biopsies were performed by the implanting transplant surgeon after reperfusion. Paraffin-embedded kidney biopsies were cut into 1–2 µm sections and stained according to a standard PAS staining protocol. Slides were digitized using Zeiss AxioScan.Z1 slide scanner (ZEISS Group, Oberkochen, Germany) with a ×20 objective and retrospectively analyzed using explainable deep-learning-based software HSA KIT (HS Analysis GmbH, Karlsruhe, Germany; Supplementary Material S1), which calculated in a reproducible and objective manner the surface area of the renal cortex and automatically quantified glomeruli. The evaluation enabled the calculation of glomerular density and the ratio of sclerosed glomeruli to the total number of glomeruli in a biopsy section (Figure 1). Histological findings of these biopsies were not available prior to transplantation and did therefore not influence decisions of the transplanting team in these patients. Data on interstitial fibrosis and tubular atrophy (IFTA), arteriolosclerosis, and arterial intimal fibrosis (AIF) were obtained from post-transplant pathology reports. Follow-up data were collected from patients undergoing routine check-up appointments at the outpatient clinic.

### Outcome Parameters

Recipient survival was defined as the time from transplantation until death, kidney graft failure by return to dialysis, excluding deaths with a functioning graft (DWFG). In the event of sepsis-induced multiple organ failure, documentation of dialysis therapy for at least 3 days prior to death was used for considering acute kidney injury as graft failure. DGF was defined as requiring more than one dialysis treatment within the first post-transplant week. Primary non-function (PNF) was defined for grafts never obtaining enough function to stop dialysis treatment after transplantation.

### Statistical Analysis

Descriptive statistics were determined for continuous (mean ± standard deviation, median, and minimum-maximum) and categorical variables (absolute values and percentages). Two-sided t-test was used to ascertain significant differences between two groups for continuous variables. Pearson’s chi-square test was applied to calculate correlations between pairs of categorical variables. The Kaplan-Meier method was employed to examine graft and patient survival and log-rank test to analyze differences in graft survival. *P*-values < 0.05 were considered to be statistically significant. The *P*-values are of descriptive nature. There was no adjustment for multiplicity. The Intraclass Correlation Coefficient (ICC) was calculated using a two-way mixed effects model with an absolute agreement model. Univariable regression analysis was conducted to determine potential prognostic factors for graft loss, PNF and DGF. Variables yielding statistical significance in the univariable analysis were evaluated through a stepwise regression process within a multivariable analysis, utilizing a binary logistic regression model. Cox proportional hazard regressions were performed univariable and multivariable in order to analyze the effect of variables on graft survival. For the multivariable model, variables with a *P*-value < 0.05 in univariable analysis were included, and backward stepwise selection was applied using a removal criterion of *P* > 0.10. All data were analyzed using SPSS 29.0 (IBM Corp., Armonk, NY, United States).

## Results

### Donor and Recipients Baseline Characteristics

A total of 64 patients who underwent kidney transplantation after ESP allocation were included in this study. All organs were obtained after brain death, as donations after circulatory death are currently not permitted in Germany. Table 1 summarizes the baseline characteristics. The mean follow-up period was 49.2 ± 16.6 months. The proportion of males was higher among both recipients (68.8%) and donors (56.3%). The mean age of the recipients was 71.3 ± 4.3 years, while the donors had a mean age of 72.9 ± 6.3 years. According to the WHO definition, male recipients showed a considerable prevalence of increased bodyweight (79.5%), compared to the overall male population in Germany within the same age group (68.2%) [8]. Mean dialysis time before transplantation was 45 months. The leading cause of renal insufficiency was hypertensive nephropathy (26.6%). The mean CIT was 8.70 ± 3.0 h, and the mean warm ischemia time (WIT) was 37.5 ± 11.5 min. Due to the missing HLA matching in the ESP, 82.8% of patients had ≥4 HLA mismatches, while only 4.7% received a full-house match.

**TABLE 1:** Demographics and clinical characteristics.

Variable	n = 64
Recipient age (years)	71.3 ± 4.3 (65–81)
Recipient sex m/f	44/20 (68.8%/31.3%)
Recipient BMI (kg/m^2^)	26.8 ± 4.06 (17.7–37.5)
Recipient Comorbidities Hypertension Coronary heart disease Diabetes Past history of tumor Renal cell cancer Prostate cancer Colorectal cancer Others	56 (87.5%) 29 (45.3%) 14 (21.9%) 21 (32.8%) 6 (9.4%) 4 (6.3%) 4 (6.3%) 7 (10.9%)
Donor age (years)	72.9 ± 6.3 (65–86)
Donor sex m/f	36/28 (56.3%/43.8%)
Donor BMI (kg/m^2^)	26.7 ± 4.8 (18.4–54.9)
Donor creatinine prior to organ procurement (mg/dL)	1.02 ± 0.50 (0.43–2.81)
Donor Comorbidities Hypertension Smoking Diabetes	34 (53.1%) 14 (21.9%) 10 (15.6%)
Time on dialysis (months)	45.0 ± 24.52 (8.72–98.69)
Renal replacement therapy HD/PD	52/12 (81.3%/18.8%)
2nd kidney transplantation	7 (10.9%)
Dual kidney transplant	3 (4.7%)
Causes for kidney failure Nephrosclerosis or hypertensive nephropathy ADPKD IgA-nephropathy Diabetic nephropathy Nephropathy of unknown case Interstitial nephritis FSGS Membranous glomerulonephritis Membranoproliferative glomerulonephritis Goodpasture-syndrome Others	17 (26.6%) 9 (14.1%) 8 (12.5%) 7 (10.9%) 4 (6.3%) 2 (3.1%) 2 (3.1%) 2 (3.1%) 1 (1.6%) 1 (1.6%) 11 (17.2%)

Data are presented as absolute values (percentages) for categorical variables; mean ± standard deviation (minimum–maximum) for continuous variables. BMI, body mass index; HD, hemodialysis; PD, peritoneal dialysis; ADPKD, autosomal dominant polycystic kidney disease; FSGS, focal segmental glomerulosclerosis.

### Predictors for Delayed Graft Function

DGF occurred in 19 out of 64 cases (29.7%). A minimal BMI disparity of ≤2.5 kg/m^2^ between donor and recipient was associated with significantly lower prevalence of DGF (11.1%), compared to >2.5 kg/m^2^ (36.9%, *P* < 0.05). Univariable analyses indicated that an unfavorable BMI match (subdivided into ≤2.5, 2.51–5.0, >5.0 kg/m^2^), higher recipient BMI, presence of CHD, and prolonged CIT significantly increased the odds of DGF. Each additional hour of CIT increased DGF-risk by 24% (*P* < 0.05). Table 2 displays the results of the uni- and multivariable analyses. In a multivariable regression model, the combination of CHD and BMI disparity reached statistical significance for the event of DGF.

**TABLE 2:** Uni- and multivariable analysis of potential risk factors for Delayed Graft Function.

Factors	Univariable analysis		Multivariable analysis	
	OR (95% CI)	P-value	OR (95% CI)	P-value
BMI match (≤2,5; 2,51–5.0; >5.0 kg/m^2^)	2.38 (1.10; 5.17)	0.028^a^	2.40 (1.07; 5.41)	0.035^a^
CHD	3.93 (1.25; 12.33)	0.019^a^	3.95 (1.20; 13.03)	0.024^a^
CIT (h)	1.24 (1.02; 1.50)	0.033^a^	--	--
Recipient BMI (kg/m^2^)	1.20 (1.03; 1.40)	0.021^a^	--	--

BMI, body mass index; BMI match, disparity in BMI between recipient and donor; CHD, coronary heart disease; CIT, cold ischemia time; OR, odds ratio; CI, confidence interval.

^a^ Significance 0.05. -- not included.

### Graft and Patient Outcome

Patients immunosuppressive therapy and outcome are described in Table 3. During the entire follow-up period, 12 patients (18.8%) died. The 1-year survival rate was 96.9%, with two patients dying within the first year and another 10 patients dying thereafter. Initially, patient survival remained nearly consistent, with a 3-year survival rate of 91.1%. After the first 3 years, the survival rate dropped, with the 5-year survival rate being only 74.0%. Seven recipients dies with a functional graft (DWFG). The primary cause of mortality was sepsis (58.3%).

**TABLE 3:** Immunosuppressive therapy, patient- and graft survival.

Variable	n = 64
HLA mismatch	4.4 ± 1.5
PRA positive recipient	12 (18.8%)
Induction therapy Basiliximab/simulect Antithymocyte globuline	60 (93.8%) 4 (6.3%)
Use of tacrolimus as initial CNI on day eight	59 (92.2%)
Use of cyclosporine A as initial CNI on day eight	5 (7.8%)
Use of an antimetabolite (MMF/MPA) on day eight	47 (73.4%)
Use of a mTOR inhibitor on day eight	17 (26.6%)
Steroid-free immunosuppression on day eight	14 (21.9%)
Delayed graft function	19 (29,7%)
Mean hospital stay after transplantation (days)	19.0 ± 8.5 (6–47)
Death	12 (18.6%)
Cause of death Sepsis Cardiovascular event Aneurysm-related hemorrhage Cancer Unknown	*n = 12* 7 (58.3%) 1 (8.3%) 1 (8.3%) 1 (8.3%) 2 (16.7%)
Graft failure	14 (21.9%)
Cause of graft failure Primary non-function As a result of infection/sepsis Rejection BK virus infection Cardiac decompensation Unknown Others	*n = 14* 5 (35.7%) 2 (14.3%) 1 (7.1%) 1 (7.1%) 1 (7.1%) 2 (14.3%) 2 (14.3%)
Duration between transplantation and graft loss (days)	617.22 ± 446.83 (89–1,177)
NODAT	11 (17.2%)
DSA	14 (21.9%)

Data are presented as absolute values (percentages) for categorical variables; mean ± standard deviation (minimum–maximum) for continuous variables. HLA, human leukocyte antigen (Loci A, B, DR); PRA, panel reactive antibodies; CNI, calcineurin-inhibitor; NODAT, new onset diabetes after transplantation; DSA, *de novo* donor-specific antibodies.

**FIGURE 2:**
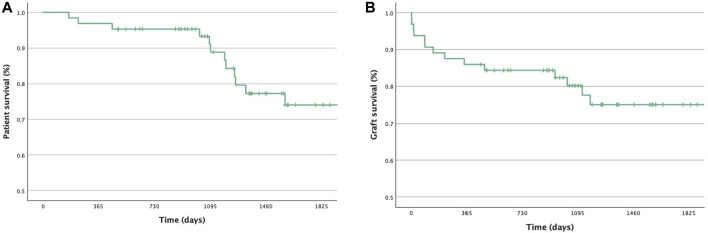
Kaplan-Meier plots for **(A)** Patient survival during follow-up. **(B)** Graft survival during follow-up (censored due to end of observation period or death with functional graft).

Graft loss occurred in 14 patients (21.9%; DWFG excluded), with 1- and 5-year graft survival rates of 85.9% and 75.0%. Kaplan-Meier curves are shown in Figures 2A,B. PNF was observed in five patients. Excluding patients with PNF, the mean time to graft failure was 617.22 ± 446.83 days (89–1,177 days). Biopsy-proven rejection was observed in 14 recipients (21.9%). However, graft loss due to chronic rejection was rare, accounting for only one case. During follow-up, DSA were identified in 14 patients (21.9%), but their presence did not correlate with graft survival or rejection events. A total of 44 patients (68.8%) were hospitalized for at least 7 days due to infection-related complications. COVID-19 was diagnosed in 15 recipients (23.4%) during one of their inpatient stays. The presence of COVID-19, BK virus infection, or cytomegalovirus did not show any statistically significant correlation with mortality or graft failure.

### Predictors for Graft Failure

Follow-up data at 4 weeks (*P* < 0.006), as well as at three (*P* = 0.039), six (*P* = 0.006) and twelve (*P* = 0.003) months after transplantation, demonstrated a statistically significant correlation between elevated creatinine levels and graft loss in univariable logistic regression model. The mean creatinine level at 4 weeks post-transplant in patients who later experienced graft failure was 3.44 mg/dL ± 1.71, compared to 2.09 ± 0.95 mg/dL in those who did not experience graft failure. Additionally, the length of hospitalization post-transplant emerged as a predictor for graft failure probability: the relative risk for the loss of a graft increased by 8% for each additional day spent in the hospital after transplantation (*P* = 0.029). As our study aimed to define parameters already available at the time of allocation, these data are presented in the Supplementary Material S2, along with factors that remained non-significant in univariate analysis and therefore were not included.

**FIGURE 3:**
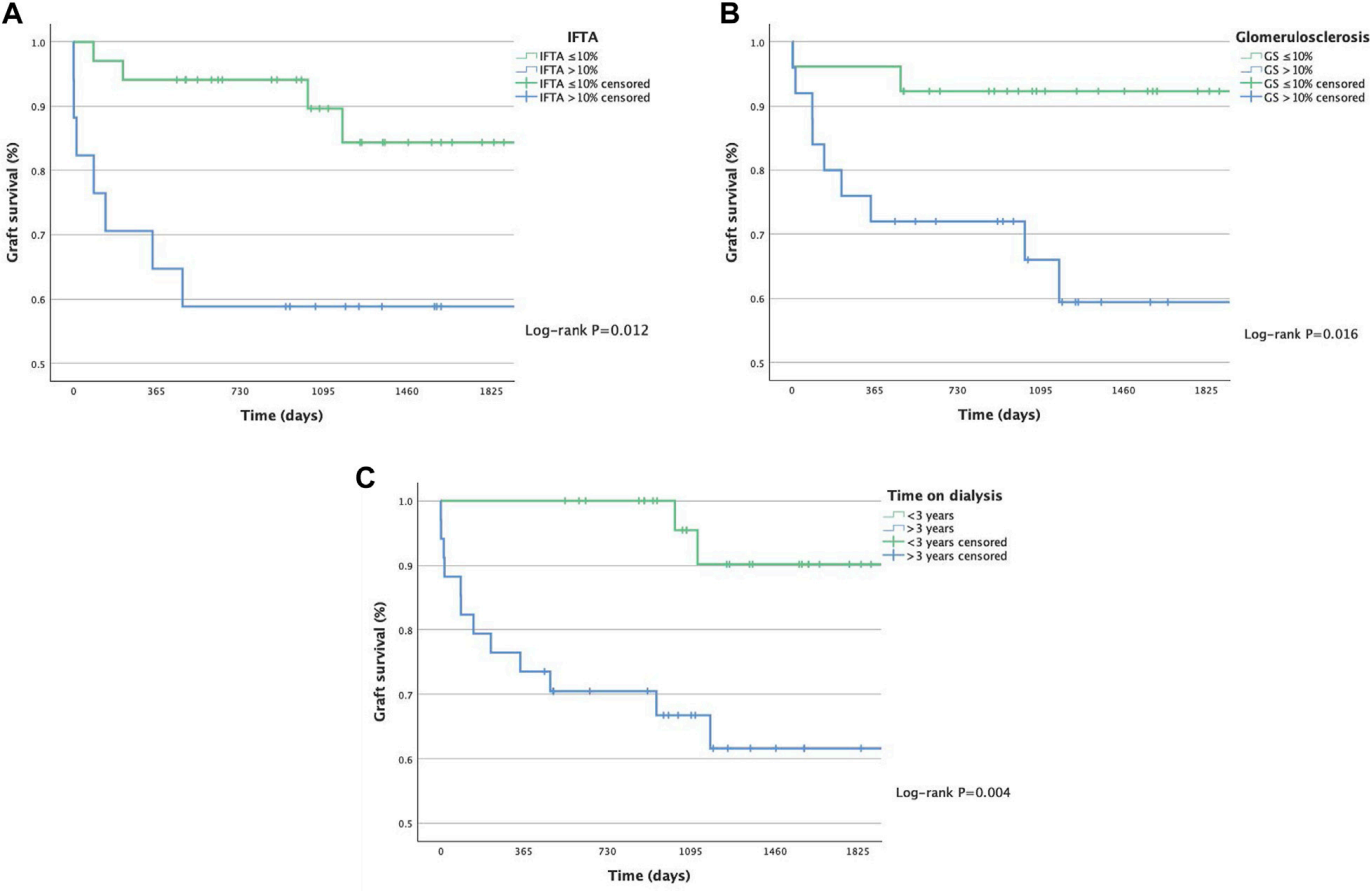
Kaplan-Meier plots for graft survival of ESP recipients by IFTA - Interstitial Fibrosis and Tubular atrophy **(A)**, degree of Glomerulosclerosis **(B)** and time on dialysis **(C)**.

Focusing on kidney donors, histopathological analysis was performed for all available 51 time-zero biopsies. There was a very good agreement on glomerulosclerosis grading between the pathologist and the retrospective semi-automated deep learning quantification (ICC = 0.913; 95% Confidence Interval = 0.85–0.95). Univariable analyses identified IFTA, the percentage of arteriolosclerosis (arteriolar hyalinosis), and glomerulosclerosis as significant risk factors for graft failure (Table 4). Glomerular density and AIF did not reach statistical significance. When focusing on the recipients, prolonged time on dialysis was associated with increased failure rates. Patients exceeding 3 years of dialysis treatment had a 35.3% risk of graft failure, compared to a 6.6% risk for those with less than 3 years of renal replacement therapy (*P* = 0.006). The combination of IFTA, glomerulosclerosis, and time on dialysis reached statistical significance in a multivariable Cox proportional hazard model. The corresponding Kaplan-Meier analyses and log-rank tests are shown in Figures 3A–C. Additionally, arteriolosclerosis showed a significant correlation for the event of PNF (*P* = 0.016; odds ratio = 1.16; 95% Confidence interval = 1.03–1.31). However, the number of HLA mismatches did not significantly influence graft survival in our ESP collective.

**TABLE 4:** Uni- and multivariable analysis of potential risk factors for graft failure using Cox Regression.

Factors	Univariable analysis		Multivariable analysis	
	HR (95% CI)	P-value	HR (95% CI)	P-value
IFTA (%)	1.04 (1.006; 1.07)	0.021^a^	1.08 (1.03; 1.41)	0.002^a^
Glomerulosclerosis (%)	1.05 (1.01; 1.09)	0.025^a^	1.07 (1.02; 1.12)	0.011^a^
Time on dialysis (months)	1.02 (1.002; 1.04)	0.031^a^	1.05 (1.02; 1.09)	0.004^a^
Arteriolosclerosis (%)	1.05 (1.01; 1.09)	0.011^a^	--	--
Arterial intima fibrosis (%)	1.01 (0.98; 1.05)	0.483	--	--
HLA-MM	0.99 (0.66; 1.50)	0.969	--	--

IFTA, Interstitial fibrosis and tubular atrophy; Glomerulosclerosis - ratio of sclerosed glomeruli to total number of glomeruli; HLA-MM, number of human leucocyte antigen mismatches; HR, Hazard ratio; CI, confidence interval.

^a^ Significance 0.05. – not included.

## Discussion

This study aimed to identify potential prognostic factors for short- and long-term outcomes of ESP-kidney transplantations to improve organ allocation strategies within the participating transplant centers in the future. Therefore, we comparatively reevaluated those parameters proposed from previous studies [1, 4, 6] for our ESP recipients and investigated potentially predictive additional variables available at the time of the organ offer, such as the matching of baseline characteristics between donors and recipients. Finally, we used deep learning based image analysis software HSA KIT as human-machine interaction tool to retrospectively quantify histopathological data obtained from time-zero kidney biopsies and its potential as a future prospective tool prior to final organ acceptance when half-automatically integrated into the allocation process.

Our univariable analysis indicated that disparity in BMI, higher recipient BMI, CHD, and prolonged CIT significantly correlated with a higher prevalence of DGF. These factors, when modifiable, may be considered in future transplant evaluations, as existing literature has demonstrated that DGF is associated with poorer outcomes [4, 9–11]. However, due to the limited size of our patient cohort, not all variables could be included in the multivariable analysis. Previous studies have consistently shown that an increased BMI in either the recipient or the donor is associated with a higher risk of DGF and graft loss [4, 12–16]. But to our knowledge, this study is the first to report the impact of BMI disparities, rather than absolute values, between donor recipient pairs within the ESP as a measure that could indeed be part of an individualized allocation decision, favoring closer BMI matches to improve outcomes, as the match might indeed guide a decision for factors (absolute BMI of donor and recipient) are non-modifiable at the time of allocation.

Analyses of time-zero biopsies revealed that histopathological findings such as IFTA and the degree of glomerulosclerosis and arteriolosclerosis represented independent predictors of graft survival in ESP recipients. Our Cox proportional hazard model points to IFTA as one of the main histological factors associated with graft survival. Ouellet et al. used IFTA scoring to demonstrate that each unit increase in IFTA at 6 months is associated with a higher risk of graft loss [17]. In this respect, it is important to emphasize that validation of AI automated IFTA scoring is still in progress at our center. Our results regarding the influence of glomerulosclerosis on graft survival as the other major histopathological determinant align with findings from other studies [18–20]. In contrast to Keijbeck et al., our observations revealed a significant association between histological arteriolosclerosis and graft outcome [21]. Much to our surprise, glomerular density and AIF were not significantly associated with graft survival, while the importance of AIF in predicting kidney function after transplantation was recently demonstrated [20].

The retrospective findings of Jacobi et al. revealed that higher biopsy scores in pre-implantation biopsies from ESP kidneys were associated with an increased prevalence of PNF and higher creatinine levels 1-year post-transplant [5]. The value of preimplantation biopsies is still a matter of debate. Given that the logistics and economics (24/7 on-call nephropathologists and technical staff), as well as the resulting time delay, would only legitimate the effort if major improvements in outcome could still be expected, considering prolonged CIT already as one of the relevant determinants of DGF and prognosis. This is where semi-automated deep learning systems could help to reduce this delay. They could be operated by the cryosectioning team (technician and pathologist), typically available at transplant centers, which are usually situated at highly specialized university hospitals. In the future, this tool may not necessarily require a designated nephropathologist during routine analysis, as only the location of the analyzed area (glomerulus, blood vessel, tubulointerstitium) needs to be validated. The agreement between retrospective semi-automated quantification and pathologist grading of glomerulosclerosis was very good [22]. However, we have not yet been able to automate the analysis of time-zero biopsies for IFTA and arteriolosclerosis. This remains a promising area for future research. Nevertheless, combining automated glomerulosclerosis-scoring with IFTA assessment by a cryosectioning on duty team might be a feasible concept today already.

In addition, a biopsy only represents a limited section of the kidney, and there may be some variation in the distribution of healthy and sclerosed glomeruli. Still, final interpretation of biopsy results needs the context of clinical and laboratory findings, although we find the opportunity of utilizing quite reliable specific parameters via deep learning systems in the environment of sparse resources very intriguing as well as applicable during our routines. Taken together, such efforts must still be justified by a significant improvement of the transplant outcomes for individual patients, considering the potential benefits of knowing histopathological details compared to the effects of procedural extension of ischemia times.

Our retrospective study was not able to confirm the positive impact of HLA-DR matching on ESP-graft survival. Fijiter et al. lately reported that HLA-DR matching for ESP-recipients resulted in reduced waiting time on dialysis (2.6 vs. 4.1 years) and improved graft survival, despite an increase in CIT (12.0 vs. 10.6 h) [1]. Furthermore, Koch et al. assert that HLA matching is even beneficial for organs from donors aged 75 and older [6]. In contrast, our findings indicate that prolonged CIT is associated with an increased risk of DGF, whereas better HLA match in our recipients did not correlate with improved outcomes. Several other studies also confirmed that extended CIT correlates with a higher incidence of DGF and graft loss [4, 11, 22, 23]. The increased susceptibility of older organs to damage from cold ischemia underscores the importance of minimizing CIT. The reduction in waiting time resulting from prospective HLA-DR matching may be the reason for better outcome, as our retrospective study again pronounces the negative impact of prolonged dialysis duration on later graft survival, as reported in the literature before [24].

DGF-rates, graft and patient survival in our study were comparable to those reported in similar studies evaluating the ESP. One- and 5-year graft survival rates ranged between 84%–87% and 63%–77%. Patient survival rates were 92%–94% and 65%–73% [4, 5, 25]. The incidence of DGF ranged between 19%–41.1% [4–6, 23, 25]. Excluding cases of PNF in our cohort, patient and graft survival rates remained stable throughout the initial 3 years, with a notable increase in mortality thereafter. Death with a functional graft occurred in 58.3% of deceased patients, which is also in line with recent ESP observations [4, 5, 16, 23]. Compared to one- and 5-year survival rates of elderly dialysis patients with end-stage kidney disease, recipients still benefited from a transplantation within the ESP. In our cohort, the 5-year survival rate for recipients aged between 65–74 years was 74.6%, as opposed to 41.0% reported for patients on dialysis [3].

In our elderly cohort of transplant recipients, sepsis was identified as the primary cause of death. This once again highlights the unmet need for individually assessed and optimized levels of immunosuppression, considering initial renal disease and immunological burden by prior immunization, immunosenescence, and the patient’s history of infections. Our results suggest that implementing less-potent immunosuppressive regimens might be advantageous, although no specific correlations of immunosuppressive therapy with patient or graft survival could be detected. In contrast to findings in previous ESP studies, in our cohort graft survival and DGF were not associated with rejection events [16]. However, the incidence of graft loss due to chronic rejection was low, and the limited number of chronic rejection cases precluded our statistical analysis from detecting potentially significant results. Taken together, follow-up care should especially evaluate the individual risk for infections and the adjustment of the immunosuppressive regimen as long as measures for individualized immunosuppressive guidance [26] cannot routinely be used.

The primary limitation of our study, next to its retrospective setup, is the relatively small sample size in terms of events for statistical testing. This constraint may have prevented identifying relationships between post-transplant outcomes and baseline characteristics such as age, diabetes mellitus, re-transplantation, and number of HLA mismatches. These factors were significant determinants of graft survival in prior ESP studies [4, 6, 16]. Our analysis of glomerular density did not yield statistically stable information regarding graft survival. An alternative approach might involve correlating glomerular density from biopsies and graft volume, which could facilitate the calculation of the total number of glomeruli in terms of “transplanted functional tissue” as a potential predictor of later transplant outcomes. These limitations could be addressed by multi-center studies with larger cohorts to prospectively validate the prognostic factors identified in this study for use during allocation. Moreover, we are quite aware that deep-learning-driven quantification would need to be validated and adapted for the use of fast-track HE-stained frozen sections, which, according to the manufacturer, would generally be technically realizable, but not yet included in our analysis.

## Data Availability

The data presented in this study are available on reasonable request by a qualified investigator for three years after the date of publication from the corresponding author.
