# Supplementary material for: Clinical and Histopathological Determinants for Kidney Allograft Survival in the Eurotransplant Senior Program (ESP) at the Time of Allocation
Source: Transpl Int. 2025 Jun 2;38:14153. doi: 10.3389/ti.2025.14153 (PMC12203019; doi:10.3389/ti.2025.14153)
Supplement: Supplementary file 1 [file DataSheet2.pdf]

Supplementary material 2

Table S1 presents variables that may have the potential to predict post-transplant course. However, these variables are not available prior to transplantation and therefore are not suitable to guide transplant decisions/ allocation acceptance. Variables that yielded statistical significance (*P*-values <0.05) in the univariable analysis were evaluated in multivariable analysis, utilizing binary logistic regression model. All data were analyzed using SPSS 29.0 (IBM Corp., Armonk, NY, USA).

Several potential prognostic factors for graft failure that were not mentioned in the main body are listed in Table S2, as they did not yield statistical significance (*P*-values <0.05) in univariable regression analysis. Therefore, these factors were not evaluated in a multivariable analysis.

Table S3 presents uni- and multivariable logistic regression analyses for factors that were significant in the univariable analysis but are only described in the main text using the proportional Cox hazard regression model for clarity.

Table S1. Uni- and multivariable analysis of post-transplant variables for graft failure

	**Univariable Analysis**		**Multivariable Analysis**	
	**OR (95% CI)**	**P-value**	**OR (95% CI)**	**P-value**
**Duration of hospitalization post-transplant** (days)	1.08 (1.01; 1.16)	0.029*	1.03 (0.94; 1.13)	0.540
**Creatinine levels after four weeks**	2.12 (1.24; 3.62)	0.006*	1.93 (1.05; 3.55)	0.034*
**Rejection episodes**	2.53 (0.68; 9.38)	0.165	--	--
**DSA**	0.22 (0.03; 1.84)	0.162	--	--

OR – odds ratio; CI – confidence interval; DSA – de-novo donor-specific antibodies.
*Significance 0.05. – not included.

Table S2. Univariable analysis of potential risk factors for graft failure

	**Univariable Analysis**	
	**OR (95% CI)**	**P-value**
**Recipient age (years)**	0.91 (0.78; 1.06)	0.228
**Donor age (years)**	1.02 (0.93; 1.11)	0.748

	Univariable Analysis	
	OR (95% CI)	P-value
**Age mismatch**	0.91 (0.76; 1.08)	0.263
**Size mismatch**	0.98 (0.90; 1.07)	0.584
**Recipient hypertension**	1.22 (0.22; 6.87)	0.819
**Recipient past history of tumor**	2.57 (0.76; 8.68)	0.128
**Recipient coronary heart disease**	0.88 (0.23; 2.91)	0.835
**Recipient diabetes mellitus**	0.63 (0.16; 2.41)	0.625
**Donor hypertension**	1.80 (0.53; 6.13)	0.347
**Donor smoking**	0.63 (0.12; 3.00)	0.588
**Donor creatinine prior to organ procurement (mg/dl)**	0.995 (0.98; 1.01)	0.522
**2nd kidney transplantation**	1.22 (0.22; 6.85)	0.819

OR – odds ratio; CI – confidence interval.

Table S3. Uni- and multivariable analysis of potential risk factors for graft failure

	Univariable Analysis		Multivariable Analysis	
	OR (95% CI)	P-value	OR (95% CI)	P-value
**IFTA** (%)	1.36 (1.02; 1.83)	0.039*	1.54 (1.07; 2.21)	0.020*
**Glomerulosclerosis** (%)	1.06 (1.01; 1.12)	0.027*	1.07 (1.003; 1.14)	0.039*
**Time on dialysis** (months)	1.03 (1.001; 1.05)	0.045*	1.05 (1.01; 1.09)	0.017*
**Arteriolosclerosis** (%)	1.05 (1.001; 1.09)	0.046*	--	--

IFTA – Interstitial fibrosis and tubular atrophy; Glomerulosclerosis - ratio of sclerosed glomeruli to total number of glomeruli; OR – odds ratio; CI – confidence interval. *Significance 0.05. – not included.
