# Supplementary material for: Clinical and Histopathological Determinants for Kidney Allograft Survival in the Eurotransplant Senior Program (ESP) at the Time of Allocation
Source: Transpl Int. 2025 Jun 2;38:14153. doi: 10.3389/ti.2025.14153 (PMC12203019; doi:10.3389/ti.2025.14153)
Supplement: Supplementary file 2 [file DataSheet1.pdf]

Supplementary Material 1

Capsule Sentence: In senior organ allocation, BMI disparities may play a relevant role for kidney-transplant success. AI -related histopathological donor-analysis at the time of allocation could further improve the prediction of the final transplant outcome in the Eurotransplant ESP-programme.

Deep Learning Method

The planning of image data experiments, creation of ground-truth data, and training of deep learning networks were completed using a deep learning-assisted workflow in the HS Analysis GmbH software. HSA STUDY manages images and data from clinical cases, while HSA AI Cockpit allows the creation or extension and validation of deep learning models without coding. All experiments and deep learning models are archived for future use. Slide quantification is automated in HSA KIT, and optimization of organ-acceptance process and improvement of allograft survival within the ESP is handled by HSA DASH. Network training in HSA AI Cockpit occurs in two stages improving validation.

In the first phase, the renal cortex is segmented and in the next step the glomeruli are detected and segmented. The whole slide images are divided into patches, which are processed by the segmentation network "HyperDeepGlomNet," a U-Net with an EfficientNet encoder. Developed by HS Analysis GmbH, HyperDeepGlomNet is a pre-trained model for glomerular segmentation, trained on diverse glomeruli data. Available in HSA KIT, it can be quickly fine-tuned with minimal training time using the HSA AI Cockpit (Figure 1).

Various deep learning experiments were done to select the performing network. Training continued for at least 60 epochs until no further improvement was seen on the validation set, which represented 10% of the dataset. All networks used a batch size of 12, and all experiments and metrics were performed in HSA AI Cockpit.

Expanding the existing foundation network “HyperDeepGlomNet” available in HSA KIT to the data from this study a comparison of two different encoders was conducted. First a EfficientNet and second a ResNet as encoders with a U-Net as decoder in each case. The results can be seen in Table [REF] and show that the EfficientNet network performs better on the validation data with an Intersection over Union of 0.9624 compared to the RestNet-based network with an Intersection over Union of 0.9512 (Table 1). Since both networks provided sufficient glomeruli segmentation, no additional experiments with different encoders or other network types, like instance segmentation models, were conducted.

The result table in HSA KIT manages experiment outcomes, providing numerically and visually traceable and reproducible quantification of biopsies on the slides, renal cortex areas and glomeruli in these areas.

Supplementary Figure Legends

Supplementary Figure 1: Flowchart of the Glomerulus Detection and Segmentation

Supplementary Table 1: Comparison of Glomerulus Segmentation Networks

Supplementary Figure 1

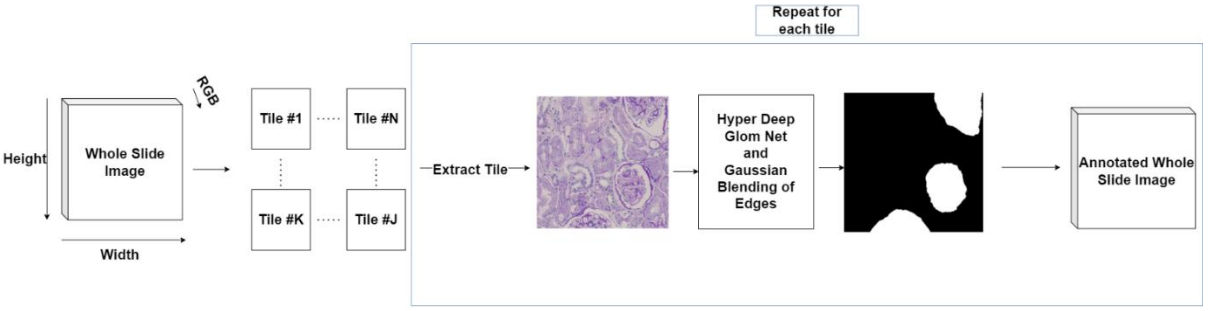

Supplementary Table 1

Model HyperDeepGlomNet	Accuracy	Intersection over Union
UNet – EfficientNet	**0.9931**	**0.9624**
UNet – ResNet	0.9846	0.9512
